# Anti-adalimumab antibodies are associated with loss of response in juvenile idiopathic arthritis

**DOI:** 10.1186/s12969-026-01213-8

**Published:** 2026-04-25

**Authors:** Mikhail Carrim, Muhammad R. A. Shipa, Aicha Bouraoui, Toka AlSulaim, Corinne Fisher, James R. W. Glanville, Maria Leandro, Debajit Sen

**Affiliations:** 1 Department of Rheumatology University College London Hospital, 250 Euston Road, London, NW1 2PG UK; 2 Department of Ageing, Rheumatology and Regenerative Medicine, Division of Medicine, University College London, London, UK; 3 Wellcome Sanger Institute, Hinxton, Saffron Walden, CB10 1RQ UK; 4 Southend University Hospital NHS Foundation Trust, Rheumatology University College London Hospitals, 250 Euston Road, London, NW1 2PG UK; 5 Rheumatology Department, King Abdulaziz Medical City, Ministry of National Guard Health Affairs, Riyadh, Saudi Arabia; 6 University College London Hospital, Department of Adolescent and Young Adult Rheumatology, 250 Euston Road, London, NW1 2PG UK

**Keywords:** Adalimumab, Anti-drug antibodies, Drug level, JIA, Loss of response, Drug failure

## Abstract

**Background:**

Secondary drug failure to adalimumab remains a significant challenge in managing Juvenile Idiopathic Arthritis (JIA), with anti-adalimumab antibodies (ADA_ab) implicated as a potential mechanism. This study examines the relationship between ADA_ab formation and loss of response (LOR).

**Findings:**

In this retrospective, longitudinal study, a combination of conventional statistical methods and machine-learning algorithms were employed to investigate the association of adalimumab drug levels (ADA_drug) and ADA_ab, alongside clinical variables, on LOR among JIA patients treated between December 2016 and May 2024. LOR was defined as active disease. Among 184 patients, ADA_ab and ADA_drug emerged as the strongest predictors of LOR in random-forest analysis. In multiple logistic regression, higher ADA_ab levels were independently associated with increased risk of LOR (OR 1.18, 95% CI 1.08–1.29, *p* = 0.0003), while higher ADA_drug levels were associated with reduced risk (OR 0.81, 95% CI 0.68–0.95, *p* = 0.0103). An ADA_ab threshold of 69 AU/mL was associated with an eightfold increase in LOR (OR 8.04, 95% CI 2.8–26.1, *p* = 0.0002). Patients with high ADA_ab and undetectable ADA_drug had the highest risk of LOR (OR 28.24, 95% CI 7.11–111.63, *p* = 0.0001). Kaplan–Meier analysis demonstrated that 71% of patients with elevated ADA_ab experienced LOR within 12 months

**Conclusion:**

High ADA_ab levels are a significant predictor of LOR in JIA patients treated with adalimumab, particularly when coupled with low drug levels. We also report clinically meaningful one-year LOR rates. These findings support the use of therapeutic drug monitoring, which may help optimise treatment and improve long-term disease control.

**Supplementary information:**

The online version contains supplementary material available at 10.1186/s12969-026-01213-8.

## Introuction

Juvenile Idiopathic Arthritis (JIA) is the most prevalent rheumatic disease of childhood. Over the past two decades, biologic therapies have transformed the treatment landscape of JIA. Despite these advances, many patients experience secondary drug failure, necessitating a switch to an alternate biologic. As these patients have aged, they have consequently cycled through successive biologic therapies, thereby diminishing the treatment options left available to them. McErlane and colleagues reported that 22% of JIA patients have been exposed to three or more biologic agents [1]. Prolonging drug survival is therefore a key goal in the long-term management of JIA. The mechanisms underlying immunogenicity and secondary failure are yet to be fully elucidated, although anti-drug antibody formation has consistently been associated with loss of therapeutic response (LOR) [2–7]. However, few large-scale longitudinal studies have investigated this association. Huang et al. [7] recently provided the first evidence of a titre dependent effect in JIA, where stronger immunogenic response towards adalimumab resulted in a greater risk of LOR. Our study builds on these findings in an independent cohort and addresses current gaps in evidence, supporting further investigation on the potential role of therapeutic drug monitoring (TDM), which may enable earlier interventions and timelier switching of therapies.

## Methods

We performed a retrospective, single-centred real-world study including longitudinal data to evaluate the impact of ADA_ab (anti-adalimumab antibodies) and ADA_drug (adalimumab drug level) on LOR (loss of response) in JIA patients treated with adalimumab (or its biosimilars) between December 2016 and May 2024 at an adolescent and young adult service at University College London Hospital. LOR was defined as ≥ 1 active joints on Juvenile Arthritis Disease Activity Score (JADAS), clinician-assessed active disease, active uveitis, synovitis on imaging, or treatment discontinuation due to flare.

Statistical analyses were conducted using RStudio (version 2024.04.2 + 764) on Mac OS platform. Missing data were managed using the MICE procedure with Markov chain Monte Carlo (MCMC) techniques under the assumption of missing at random (MAR) [8, 9] (Additional Methods 1). After evaluating multiple machine learning algorithms, the top-performing models—partial least squares regression (sPLS-DA), regularised random forest (RRF), and support vector machines (SVM)-were selected. These models were validated using cross-validation, with their performance assessed through confusion matrices, F1 scores, and accuracy metrics. Continuous variables were standardised by centering and scaling for the machine learning algorithm. Key predictors were identified through variable selection methods and further analysed using logistic regression, with odds ratios (ORs) and 95% confidence intervals (CIs) reported. Propensity score adjustment was employed in logistic regression to control for potential confounders such as age, gender, and clinical characteristics. For continuous variables, where applicable, optimal cut-points were determined using AUROC analysis. Time-to-event data were analysed using Kaplan–Meier estimators and Cox regression to assess hazard ratios (HRs). Longitudinal changes were examined using a linear mixed-effects model, with adjustments made for relevant covariates.

## Results

Among our cohort, 184 individuals treated with adalimumab had recorded measurements for both ADA_drug and ADA_ab (Additional Table 3). The majority of patients identified as white (60%), followed by mixed/other (28%), Asian (10%), and black (3%). The median age of JIA onset among our cohort was 9 years (IQR 3–13). The median age at evaluation was 20 years. Females comprised 56% of the cohort, and 62% of patients were receiving concomitant disease-modifying antirheumatic drugs (DMARDs). Enthesitis-Related Arthritis (ERA) was the most common JIA subtype (36%).

**Fig. 1:**
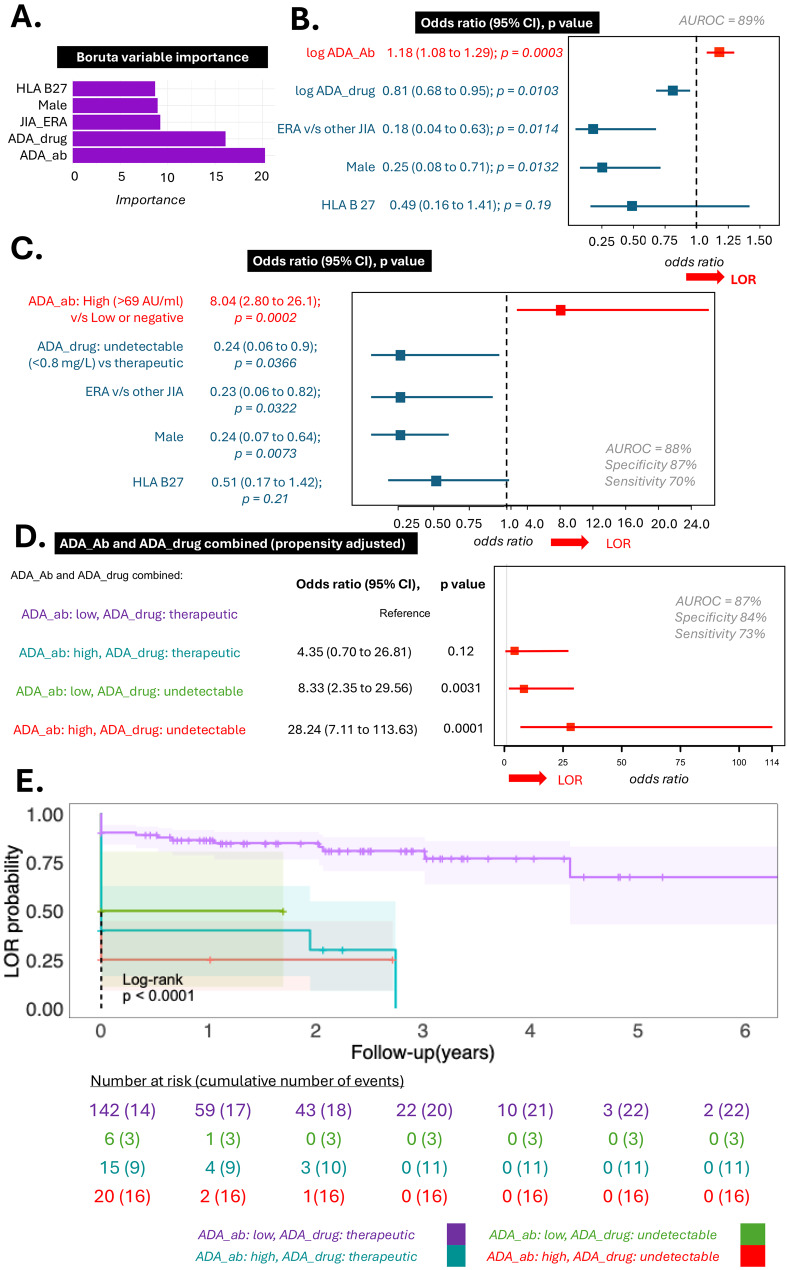
**A–E** | Factors influencing loss of remission (LOR) among Juvenile Idiopathic Arthritis (JIA) patients treated with adalimumab (total *n* = 184; LOR *n* = 52). **A** Boruta random-forest feature selection showing variable importance (Boruta Z-scores, randomForest Mean Decrease Accuracy). Each predictor’s importance was compared against permuted “shadow” features, and Bonferroni-adjusted *p*-values were used to classify variables as confirmed important. **B** Final multiple logistic regression using these five variables, showing odds ratios (ORs), 95% confidence intervals (CIs), *p*-values, and area under the receiver operating characteristic curve (AUROC). ADA_ab and ADA_drug were log-transformed. **C** Final multiple logistic regression using these five variables, with anti-adalimumab antibodies (ADA_ab; AU/mL) and adalimumab drug levels (ADA_drug; ng/mL) stratified into two groups. The optimal ADA_ab threshold was 69 AU/mL. Because ADA_drug showed a bimodal distribution and unstable bootstrap-derived cut-points, the manufacturer’s recommended threshold of 0.8 ng/mL was used (values > 0.8 ng/mL deemed therapeutic). **D** Propensity-score† adjusted multiple logistic regression assessing the combined effects of ADA_ab and ADA_drug on LOR. Patients were categorised into four groups based on ADA_ab (high/low) and ADA_drug (therapeutic/undetectable). **E** Kaplan–Meier (K–M) survival curves illustrating time to LOR for these four groups, with the table below showing numbers at risk and cumulative events. ^Missing data were managed using the MICE procedure, employing Markov chain Monte Carlo (MCMC) methods under the assumption of missing at random (MAR). ^†^ The propensity score was estimated for each patient via logistic regression adjusted for age, sex, ethnicity, disease duration, JIA subtype, concomitant DMARDs (disease-modifying antirheumatic drugs), ESR (erythrocyte sedimentation rate), CRP (C-reactive protein), presence of uveitis, psoriasis, or inflammatory bowel disease, HLA B27 status, and antibody status

Using a Random-Forest algorithm, ADA_ab and ADA_drug emerged as the variables most significantly associated with LOR, followed by JIA subtype (notably ERA), male sex, and HLA-B27 status (Fig. 1A). Univariate baseline comparisons are shown in Additional Table 1 (imputed) and Additional Table 2 (un-imputed). Sensitivity analyses were consistent across approaches: ADA_ab levels were significantly higher in patients with LOR than those without LOR (median 66 AU/mL [IQR 0–200] vs 0 AU/mL [IQR 0–0], *p* < 0.0001), while ADA_drug levels were significantly lower (median 2.2 ng/mL [IQR 0–5.2] vs 10.6 ng/mL [IQR 7.6–14.0], *p* < 0.0001). On univariate analysis, baseline ADA_ab positivity, defined using the manufacturer’s threshold (≥6 AU/mL), was significantly more frequent among patients who developed LOR compared with those who remained in remission (36/52 [69%] vs 25/132 [19%], *p* < 0.0001). In a multiple logistic regression (MLR) model, higher ADA_ab levels emerged as the only significant predictor of increased LOR risk (OR 1.18, 95% CI 1.08–1.29, *p* = 0.0003), whereas higher ADA_drug levels were associated with lower LOR risk (OR 0.81, 95% CI 0.68–0.95, *p* = 0.0103) (Fig. 1B).

We then sought optimal cut-points for ADA_ab (AU/mL) and ADA_drug (ng/mL) using bootstrap AUROC analysis. For ADA_ab, the optimal threshold was 69 AU/mL. Using this threshold, baseline ADA_ab positivity was seen in 26/52 (50%) patients who developed LOR compared with 9/132 (7%) patients who remained in remission (*p* < 0.0001). For ADA_drug, the distribution was bimodal, with a large cluster of results at 0 ng/mL (undetectable) and a second cluster at higher concentrations (therapeutic range), resulting in unstable bootstrap-derived cut-points; therefore, we used the manufacturer-recommended threshold of 0.8 ng/mL to define undetectable versus therapeutic drug levels. When categorised using these thresholds, elevated ADA_ab remained strongly associated with LOR (OR 8.04, 95%CI 2.8–26.1, *p* = 0.0002) (Fig. 1C).

Next, we examined the combined effect of ADA_ab and ADA_drug by defining four groups: (i) ADA_ab: high, ADA_drug: therapeutic (16 patients), (ii) ADA_ab: high, ADA_drug: undetectable (19 patients), (iii) ADA_ab: low, ADA_drug: therapeutic (143 patients), (iv) ADA_ab: low, ADA_drug: undetectable (6 patients). Groups (i) and (ii) demonstrated higher LOR rates, with propensity-adjusted odds ratios of 8.33 (95%CI 2.35–29.56, *p* = 0.0030) and 28.24 (95%CI 7.11–111.63, *p* = 0.0001), respectively (Fig. 1D).

Kaplan–Meier analysis demonstrated a shorter time to LOR in patients with high ADA_ab levels, regardless of ADA_drug status, compared with the ‘ideal response’ group of patients with low ADA_ab and therapeutic ADA_drug (log-rank *p* < 0.0001; Fig. 1E). Pairwise Cox regression further showed that high ADA_ab was associated with a higher hazard of LOR over time irrespective of ADA_drug status. Compared with the “ideal response” group, the hazard of LOR was significantly higher in patients with high ADA_ab and undetectable ADA_drug (HR 10.34, 95% CI 5.28–20.29, *p* < 0.0001) and remained higher in those with high ADA_ab despite therapeutic ADA_drug levels (HR 5.79, 95% CI 2.74–12.18, *p* < 0.0001). Notably, 71% of patients with high ADA_ab experienced LOR within 12 months.

## Discussion

At the time of writing, this is the largest single centred, longitudinal study investigating the association between ADA_ab and ADA_drug on disease activity in JIA. These findings validate the titre-dependent association described by Huang et al. [7] and replicate it in an independent cohort. We identified a clinically relevant cut-off antibody threshold of 69 AU/mL, above which there is an approximately eightfold increased risk of LOR. The longitudinal design further demonstrates that a significant proportion of patients with high ADA_ab levels were observed to experience LOR in one year, thus highlighting the potential value of regular antibody monitoring. This can help clinicians identify patients who may benefit from closer follow-up or the addition of a DMARD to prolong drug survival [5, 7]. This is particularly impactful in paediatrics and adolescents, where JIA manifests early in life and treatment options remain limited. In this context, prolonged biologic survival not only preserves therapeutic benefit but also safeguards the limited pool of biologics available over a patient’s lifetime. Alternatively, high ADA_ab may also help guide the decision for biologic switch.

A key strength of our research is its scale: to our knowledge it is the largest real-world study evaluating ADA_ab and ADA_drug levels in JIA, providing enhanced statistical power. This, coupled with our robust methodology, combining machine learning and traditional statistical models strengthens confidence in our findings. The concurrent measurement of adalimumab drug levels allows us to demonstrate that both ADA_ab and ADA_drug are independently associated with LOR, and when combined are associated with substantially higher odds of LOR. This supports the potential role of TDM in this population.

Limitations include the retrospective design and non-standardised sampling times, potentially limiting the accuracy of true trough measurements. Assay variability across centres may also affect generalisability of our proposed cut-offs. However, given the limited number of laboratories performing this investigation, and the growing body of evidence supporting the association between ADA_ab, ADA_drug and LOR, assay standardisation should be achievable. This could ultimately support the establishment of nationally recognised guidelines. Finally, low ADA_drug levels may reflect poor adherence rather than immunogenicity.

Although our results demonstrate a robust correlation, prospective studies are needed to validate these findings and determine their implications for clinical guidelines, while pharmacokinetic and pharmacodynamic studies will be needed to provide greater insight into the precise aetiology of immunogenicity towards adalimumab and its relationship with LOR.

## Conclusion

This study provides the most comprehensive real-world evidence to date linking ADA_ab levels with loss of response in JIA. We replicate the findings of Huang et al. [7] and extend them through a larger sample size, robust statistical and machine learning models, and by demonstrating clinically meaningful one-year LOR rates in patients with high ADA_ab. Our findings indicate the potential role of TDM to guide JIA management, enabling early intervention and personalised treatment strategies.

## Electronic supplementary material

Below is the link to the electronic supplementary material.

Supplementary Material 1

## Data Availability

Fully Anonymised individual patient data will be shared upon reasonable request for research purposes.

## Abbreviations

JIA: Juvenile idiopathic arthritis
ADA_ab: Anti-adalimumab antibodies
LOR: Loss of response
ADA_drug: Adalimumab drug levels
TDM: Therapeutic drug monitoring
JADAS: Juvenile arthritis disease activity score
MCMC: Markov chain Monte Carlo
MAR: Missing at random
ORs: Odds ratios
CIs: Confidence intervals
HRs: Hazard ratios
DMARDs: Disease-modifying antirheumatic drugs
ERA: Enthesitis-related arthritis

## Code availability

Code will also be shared upon reasonable request.

## References

[1] McErlane F, Foster HE, Davies R, Lunt M, Watson KD, et al. Biologic treatment response among adults with juvenile idiopathic arthritis: results from the British society for rheumatology biologics Register. Rheumatol (Oxford). 2013, Oct;52(10):1905–13. 10.1093/rheumatology/ket248. 10.1093/rheumatology/ket248 PMC3775296 23873820

[2] Skrabl-BaumgartnerA, Erwa W, Muntean W, Jahnel J. Anti-adalimumab antibodies in juvenile idiopathic arthritis: frequent association with loss of response. Scand J Rheumatol. 2015;44(5). 10.3109/03009742.2015.1022213. 10.3109/03009742.2015.1022213 25974288

[3] Marino A, Real-Fernández F, Rovero P, Giani T, Pagnini I, Cimaz R, et al. Anti-adalimumab antibodies in a cohort of patients with juvenile idiopathic arthritis: incidence and clinical correlations. Clin Rheumatol. 2018;37(5). 10.1007/s10067-018-4057-7 10.1007/s10067-018-4057-7 29508177

[4] Doeleman MJH, De Roock S, El Amrani M, Van Maarseveen EM, Wulffraat NM, Swart JF. Association of adalimumab trough concentrations and treatment response in patients with juvenile idiopathic arthritis. Rheumatol (United Kingdom). 2022;61(1). 10.1093/rheumatology/keab35 10.1093/rheumatology/keab354 33878159

[5] Doeleman MJH, Van Maarseveen EM, Swart JF. Immunogenicity of biologic agents in juvenile idiopathic arthritis: a systematic review and meta-analysis. Rheumatol (United Kingdom). 2019;58(10). 10.1093/rheumatology/kez030 10.1093/rheumatology/kez030 PMC6758589 30809664

[6] Brunelli JB, Silva CA, Pasoto SG, Saa CGS, Kozu KT, Goldenstein-Schainberg C, et al. Anti-adalimumab antibodies kinetics: an early guide for juvenile idiopathic arthritis (JIA) switching. Clin Rheumatol. 2020;39(2). 10.1007/s10067-019-04798-6 10.1007/s10067-019-04798-6 31707543

[7] Huang BH, Hsu JL, Huang HY, Huang JL, Yeh KW, Chen LC, et al. Early Anti-drug antibodies predict adalimumab response in juvenile idiopathic arthritis. Int J Mol Sci. 2025, Jan, 30;26(3):1189. 10.3390/ijms26031189 39940955 10.3390/ijms26031189 PMC11818047

[8] White IR, Royston P, Wood AM. Multiple imputation using chained equations: issues and guidance for practice. Stat Med. 2011;30(4). 10.1002/sim.4067 21225900

[9] Resche-Rigon M, White IR. Multiple imputation by chained equations for systematically and sporadically missing multilevel data. Stat Methods Med Res. 2018;27(6). 10.1177/0962280216666564 PMC5496677 27647809

