# Supplementary material for: Anti-adalimumab antibodies are associated with loss of response in juvenile idiopathic arthritis
Source: Pediatr Rheumatol Online J. 2026 Apr 25;24:37. doi: 10.1186/s12969-026-01213-8 (PMC13273996; doi:10.1186/s12969-026-01213-8)
Supplement: Supplementary file 1 — Supplementary Material 1 [file 12969_2026_1213_MOESM1_ESM.docx]

**Anti-Adalimumab Antibodies are Associated with Loss of Response in Juvenile Idiopathic Arthritis**

**Authors:** Mikhail Carrim^1^, Muhammad RA Shipa^2^, Aicha Bouraoui^3^ Toka AlSulaim^4^ Corinne Fisher^5^, James R W Glanville^6^, Maria Leandro^7^, Debajit Sen^8^

**Additional Tables:**

| **Additional Table 1 \| Univariate logistic regression on imputed dataset (predicting LOR/loss of remission)^1^** | | | | | |
| --- | --- | --- | --- | --- | --- |
| **Characteristic** | **N** | **Event N** | **OR^2^** | **95% CI***^2^* | **p-value** |
| **Age** | 184 | 52 | 0.77 | 0.48, 1.11 | 0.18 |
| **Sex** | 184 | 52 |  |  | **<0.001** |
| Female |  |  | — | — |  |
| Male |  |  | 0.20 | 0.09, 0.43 |  |
| **Ethnicity** | 184 | 52 |  |  | 0.17 |
| White |  |  | — | — |  |
| Black |  |  | 0.00 |  |  |
| Asian |  |  | 0.53 | 0.12, 1.76 |  |
| Others |  |  | 1.23 | 0.60, 2.50 |  |
| **CRP**  **(standardised)** | 184 | 52 | 1.46 | 1.03, 2.35 | **0.030** |
| **ESR**  **(standardised)** | 184 | 52 | 2.02 | 1.45, 2.88 | **<0.001** |
| **ADA_drug**  **(standardised)** | 184 | 52 | 0.24 | 0.15, 0.38 | **<0.001** |
| **ADA_ab**  **(standardised)** | 184 | 52 | 2.87 | 2.03, 4.24 | **<0.001** |
| **cDMARD** | 184 | 52 |  |  | 0.31 |
| None |  |  | — | — |  |
| MTX |  |  | 0.55 | 0.28, 1.10 |  |
| SSZ |  |  | 0.59 | 0.12, 2.18 |  |
| Others (Mycophenolate, HCQ, leflunomide) |  |  | 0.39 | 0.06, 1.67 |  |
| **Dose** | 184 | 52 | 0.79 | 0.43, 1.15 | 0.27 |
| **Route** | 184 | 52 |  |  | 0.28 |
| oral |  |  | — | — |  |
| Subcutaneous |  |  | 0.70 | 0.37, 1.34 |  |
| **HLAB27** | 184 | 52 |  |  | **<0.001** |
| Negative |  |  | — | — |  |
| Positive |  |  | 0.23 | 0.10, 0.49 |  |
| **ANA** | 184 | 52 |  |  | **0.001** |
| Negative |  |  | — | — |  |
| Positive |  |  | 3.00 | 1.55, 5.87 |  |
| **RF** | 184 | 52 |  |  | 0.17 |
| Negative |  |  | — | — |  |
| Positive |  |  | 0.60 | 0.28, 1.23 |  |
| **CCP** | 184 | 52 |  |  | 0.38 |
| Negative |  |  | — | — |  |
| Positive |  |  | 0.73 | 0.36, 1.45 |  |
| **JIA** | 184 | 52 |  |  | **<0.001** |
| Others |  |  | — | — |  |
| Enthesitis related arthritis |  |  | 0.13 | 0.05, 0.34 |  |
| **Uveitis** | 184 | 52 |  |  | 0.63 |
| Yes |  |  | — | — |  |
| No |  |  | 0.83 | 0.38, 1.73 |  |
| **Psoriasis** | 184 | 52 |  |  | 0.86 |
| Yes |  |  | — | — |  |
| No |  |  | 1.08 | 0.42, 2.58 |  |
| **IBD** | 184 | 52 |  |  | **0.005** |
| Yes |  |  | — | — |  |
| No |  |  | 0.12 | 0.01, 0.59 |  |
| *^1^ Continuous variables were standardised by centering and scaling*  *^2^ OR = Odds Ratio, CI = Confidence Interval* | | | | | |

| **Additional Table 2 \| Univariate logistic regression on un-imputed dataset (predicting LOR/loss of remission)^1^** | | | | | |
| --- | --- | --- | --- | --- | --- |
| **Characteristic** | **N** | **Event N** | **OR^2^** | **95% CI^2^** | **p-value** |
| **Age** | 184 | 52 | 0.77 | 0.48, 1.11 | 0.18 |
| **Sex** | 184 | 52 |  |  | **<0.001** |
| Female |  |  | — | — |  |
| Male |  |  | 0.20 | 0.09, 0.43 |  |
| **Ethnicity** | 184 | 52 |  |  | 0.17 |
| White |  |  | — | — |  |
| Black |  |  | 0.00 |  |  |
| Asian |  |  | 0.53 | 0.12, 1.76 |  |
| Others |  |  | 1.23 | 0.60, 2.50 |  |
| **CRP (standardised)** | 171 | 48 | 1.43 | 1.01, 2.31 | **0.042** |
| **ESR (standardised)** | 171 | 49 | 2.09 | 1.08, 3.05 | **0.024** |
| **ADA_drug**  **(standardised)** | 183 | 52 | 0.38 | 0.21, 0.62 | **<0.001** |
| **ADA_ab**  **(standardised)** | 183 | 51 | 2.87 | 1.99, 4.12 | **<0.001** |
| **cDMARD** | 176 | 44 |  |  | 0.54 |
| None |  |  | — | — |  |
| MTX |  |  | 0.58 | 0.44, 1.43 |  |
| SSZ |  |  | 0.60 | 0.29, 3.22 |  |
| Others (Mycophenolate, HCQ, leflunomide) |  |  | 0.43 | 0.06, 1.82 |  |
| **Dose** |  |  | 0.48 | 0.07, 2.10 |  |
| **Route** | 175 | 47 | 0.66 | 0.24, 1.08 | 0.12 |
| oral | 70 | 17 |  |  | 0.44 |
| Subcutaneous |  |  | — | — |  |
| **HLAB27** |  |  | 0.63 | 0.18, 1.99 |  |
| Negative | 117 | 31 |  |  | **0.006** |
| Positive |  |  | — | — |  |
| **ANA** |  |  | 0.28 | 0.09, 0.70 |  |
| Negative | 127 | 41 |  |  | **0.007** |
| Positive |  |  | — | — |  |
| **RF** |  |  | 2.95 | 1.35, 6.53 |  |
| Negative | 117 | 37 |  |  | 0.74 |
| Positive |  |  | — | — |  |
| **CCP** |  |  | 0.79 | 0.17, 2.94 |  |
| Negative | 95 | 31 |  |  | 0.52 |
| Positive |  |  | — | — |  |
| **JIA** |  |  | 0.50 | 0.02, 3.57 |  |
| Others | 183 | 51 |  |  | **<0.001** |
| Enthesitis related arthritis |  |  | — | — |  |
| **Uveitis** |  |  | 0.13 | 0.05, 0.35 |  |
| Yes | 184 | 52 |  |  | 0.63 |
| No |  |  | — | — |  |
| **Psoriasis** |  |  | 0.83 | 0.38, 1.73 |  |
| Yes | 184 | 52 |  |  | 0.86 |
| No |  |  | — | — |  |
| **IBD** |  |  | 1.08 | 0.42, 2.58 |  |
| Yes | 184 | 52 |  |  | **0.005** |
| No |  |  | — | — |  |
| 1 |  |  | 0.12 | 0.01, 0.59 |  |
| *^1^ Continuous variables were standardised by centering and scaling*  *^2^ OR = Odds Ratio, CI = Confidence Interval* | | | | | |

| **Additional Table 3 \| Baseline characteristics** | |
| --- | --- |
| **Characteristic** | **N = 184***^1^* |
| Age*^1^* | 20.0 (18.0, 25.0) |
| Sex |  |
| Female | 103 (56%) |
| Male | 81 (44%) |
| Ethnicity (reported by patients) |  |
| White | 111 (60%) |
| Black | 5 (2.7%) |
| Asian | 17 (9.2%) |
| Others | 51 (28%) |
| CRP*^1^* | 1.0 (0.0, 2.4) |
| Missing | 13 |
| ESR*^1^* | 5 (2, 12) |
| Missing | 13 |
| ADAD*^1^* | 9.3 (3.4, 13.1) |
| Missing | 1 |
| ADAA*^1^* | 0 (0, 22) |
| DMARDs (Disease modifying anti-rheumatic drugs) |  |
| None | 67 (38%) |
| Methotrexate | 88 (50%) |
| Sulfasalazine | 11 (6.3%) |
| Mycophenolate | 3 (1.7%) |
| Azathioprine | 3 (1.7%) |
| 6-Mercaptopurine | 2 (1.1%) |
| Hydroxychloroquine | 1 (0.6%) |
| Leflunomide | 1 (0.6%) |
| Missing | 8 |
| Route |  |
| Oral | 44 (63%) |
| Subcutaneous | 26 (37%) |
| HLAB27 |  |
| Negative | 71 (61%) |
| Positive | 46 (39%) |
| Missing | 67 |
| ANA |  |
| Negative | 87(65%) |
| Positive | 46 (35%) |
| Missing | 51 |
| RF |  |
| Negative | 106 (90%) |
| Positive | 12 (10%) |
| Missing | 66 |
| CCP |  |
| Negative | 90 (95%) |
| Positive | 5 (5%) |
| Missing | 89 |
| JIA |  |
| Persistent Oligoarticular JIA | 22 (12%) |
| Extended Oligoarticular JIA | 14 (7.7%) |
| Polyarticular JIA | 47 (26%) |
| Systemic JIA | 1 (0.5%) |
| Enteropathy associated JIA | 11 (6.0%) |
| Enthesitis related arthritis | 65 (36%) |
| Psoriasis associated JIA | 21 (11%) |
| Chronic uveitis | 2 (1.1%) |
| Missing | 1 |
| Uveitis | 47 (26%) |
| Psoriasis | 27 (15%) |
| IBD | 20 (11%) |
| ADA_ab |  |
| Negative | 123 (67%) |
| Positive | 61 (33%) |
| *^1^* Median (Q1, Q3); n (%) | |

**Additional methods**

Missing data were managed using the multivariate imputation by chained equations (MICE) procedure with Markov chain Monte Carlo (MCMC) techniques under the assumption of missing at random (MAR) (8,9). This assumption was considered reasonable as missingness largely reflected variation in routine clinical sampling and documentation and was plausibly related to observed patient/disease characteristics. The MICE algorithm iteratively generated 50 complete datasets. Imputation models were specified according to variable type (predictive mean matching for continuous variables and logistic/multinomial regression for categorical variables). The imputation model included the outcome (LOR) and all covariates used in subsequent analyses. Analyses were performed within each imputed dataset and pooled using Rubin’s rules^1^.

Machine-learning was used as an exploratory variable-selection step to inform conventional multivariable modelling and mitigate the impact of collinearity/correlation and potential non-linear relationships/interactions. Candidate algorithms were compared using 10-fold cross-validation (confusion matrices, accuracy and F1 score), and regularised random forest showed the best performance (accuracy 85%, F1=0.85). For the RRF model, hyperparameters were tuned within cross-validation, including the number of trees (ntree, evaluated across a range up to 10,001 to ensure stability), mtry (number of variables sampled at each split) and maxnode (maximum terminal nodes), with the final settings selected based on best cross-validated performance. Important predictors were identified using Boruta and carried forward into the primary inferential analysis, multivariable logistic regression, reporting odds ratios (ORs) with 95% confidence intervals (CIs). Results from both imputed and complete-case multiple logistic regression models are presented (Supplementary Table 1-2). Continuous predictors were mean-centred and SD-scaled for the machine-learning workflow only. ADA_ab and ADA_drug were log-transformed.

Propensity score (PS) adjustment was used to reduce confounding when comparing outcomes across four exposure groups defined by ADA_ab (high/low) and ADA_drug (therapeutic/undetectable). The PS model included age, sex, ethnicity, disease duration, JIA subtype, concomitant DMARD use, ESR, CRP, uveitis, psoriasis, inflammatory bowel disease, HLA-B27, and antibody status. Covariate balance was assessed using standardised mean differences before and after adjustment. The PS was incorporated by including it as an adjustment covariate in the logistic regression model for the four-group comparison. Where applicable, optimal cut-points for continuous variables were determined using AUROC analysis with bootstrap resampling (1,000 iterations) and selection of the threshold that maximised Youden’s index. Time-to-event data were analysed using Kaplan–Meier estimators and Cox regression to estimate hazard ratios (HRs) with 95% confidence intervals.

Serum adalimumab drug levels and anti-adalimumab antibodies were measured using a drug-tolerant enzyme-linked immunosorbent assay (ELISA) detecting total anti-adalimumab antibodies (Exeter Blood Sciences Laboratory, United Kingdom), performed according to the manufacturer’s instructions^2^.

**References**

1. Rubin, D.B. (1987) Multiple Imputation for Nonresponse in Surveys. John Wiley & Sons Inc., New York. http://dx.doi.org/10.1002/9780470316696
2. Exeter Clinical Laboratory International. Blood Science Tests. Adalimumab Antibody Levels. Available at: [https://www.exeterlaboratory.com/test/adalimumab-antibody-levels/.](https://www.exeterlaboratory.com/test/adalimumab-antibody-levels/)
